# Association between NT-proBNP Level and the Severity of COVID-19 Pneumonia

**DOI:** 10.1155/2021/5537275

**Published:** 2021-07-08

**Authors:** Lan Wang, Fei Chen, Lin Bai, Lang Bai, Zhixin Huang, Yong Peng

**Affiliations:** ^1^Department of Respiratory and Critical Care Medicine, West China Hospital, Sichuan University, Chengdu, China; ^2^Medical Team from West China Hospital of Sichuan University in Hubei General Hospital (East Branch), Wuhan, China; ^3^Department of Cardiology, West China Hospital, Sichuan University, Chengdu, China; ^4^Infectious Disease Center, West China Hospital, Sichuan University, Chengdu, China; ^5^Hubei General Hospital (East Branch) & Renmin Hospital of Wuhan University, Wuhan, China

## Abstract

In this study, we investigated the association between the plasma NT-proBNP level at admission and the severity of COVID-19 pneumonia. For this retrospective, single-centre cohort study, we enrolled consecutive patients from February 9 to March 4, 2020, in a COVID-19 ward of Hubei General Hospital (East Branch) in Wuhan, which is a government-assigned centre for COVID-19 treatment. Diagnosis was confirmed by microbiological and radiographic findings following the interim guidance of the World Health Organization (WHO). A total of 91 (92.9%) patients were finally included in this study. The median age of the patients was 61 years (IQR, 47–69), and 39 (43.0%) of them were male. Two cases of death were reported (2.3%). Twenty-three patients (25.3%) had NT-proBNP levels above 300 pg/ml. Higher NT-proBNP levels were associated with worse PSI and CT scores. The natural logarithm of the NT-proBNP level was positively correlated with the PSI and CT scores (PSI score: *r*_*S*_ = 0.396, *P*=0.001; CT score: *r*_*S*_ = 0.440, *P* < 0.001). Patients with NT-proBNP ≥300 pg/ml showed a potential risk for higher mortality than patients with NT-proBNP <300 pg/ml (mortality rate, 8.7% vs. 0%; *P*=0.062). The plasma NT-proBNP level of COVID-19 patients was significantly related to the severity of pneumonia.

## 1. Introduction

Recently, severe acute respiratory syndrome coronavirus 2 (SARS-CoV-2) has spread rapidly and has attracted attention given the gravity of the situation. Since the outbreak of coronavirus disease 2019 (COVID-19), cardiovascular complications have become an important cause of deterioration and death in many patients [1, 2]. Cardiovascular complications of acute pneumonia, including myocarditis and exacerbation of cardiac insufficiency, have been well recognized during previous historical epidemics [3]. An increase in the level of N-terminal pro-B-type natriuretic peptide (NT-proBNP) indicates impairment of cardiac function and is an important biomarker for the diagnosis and estimation of prognosis in cardiac insufficiency. In the current study, we aimed to investigate the association between the plasma NT-proBNP level at admission and the severity of COVID-19 pneumonia.

## 2. Methods

### 2.1. Study Population

**Figure 1:**
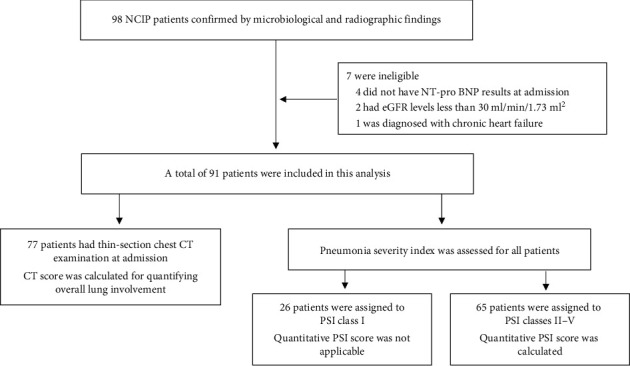
Flowchart of patient inclusion. NT-proBNP: N-terminal pro-B-type natriuretic peptide; eGFR: estimated glomerular filtration rate; HRCT: high-resolution computed tomography; PSI: pneumonia severity index.

For this retrospective, single-centre cohort study, we enrolled consecutive patients from February 9 to March 4, 2020, at a COVID-19 ward (with a medical assistance team from West China Hospital) in Hubei General Hospital (East Branch) in Wuhan, China, which was assigned by the government and was responsible for the treatment of patients with COVID-19. COVID-19 pneumonia was diagnosed based on the interim guidance of the World Health Organization (WHO) [4]. Laboratory-confirmed cases were defined by a positive result on a real-time reverse-transcriptase polymerase chain reaction (RT-PCR) assay of nasal and/or pharyngeal swabs. Only cases confirmed by microbiological and radiographic findings were eligible for inclusion. We excluded patients with chronic heart failure, severe chronic kidney disease (eGFR <30 ml/min/1.73 m^2^), or those who did not have NT-proBNP results at admission (the flowchart of patient inclusion is presented in Figure 1). Demographic data, medical history, vital signs at admission, laboratory tests, and radiographic results were obtained from the patients' electronic medical records and reviewed by two independent researchers.

### 2.2. PSI

We used the pneumonia severity index (PSI) to quantify disease severity [5]. Briefly, patients who met the following criteria were defined as class I: (1) age ≤50; (2) no history of any of the following coexisting conditions: neoplastic disease, congestive heart failure, cerebrovascular disease, renal disease, or liver disease; and (3) none of the following abnormalities on physical examination: altered mental status, pulse ≥125/minute, respiratory rate ≥30/minute, systolic blood pressure <90 mmHg, or temperature <35°C or ≥40°C. The other patients were assigned to risk classes II–V according to their clinical characteristics (the flowchart for the pneumonia severity index is provided in Supplementary Appendix I, Figure S1).

### 2.3. CT Score

Attending physicians in respiratory medicine reviewed the high-resolution chest computed tomography (CT) images to compute the CT scores, which were used to quantify the overall lung involvement [6]. Briefly, thin-section CT imaging scans were reviewed by attending physicians in respiratory medicine. Ground-glass opacities (GGOs) were defined as hazy areas of increased opacity or attenuation without concealing the underlying vessels. Consolidation was defined as homogeneous opacification of the parenchyma with obscuration of the underlying vessels. The presence of parenchymal bands, irregular linear opacities, interfaces, and traction bronchiectasis was considered evidence of probable fibrosis. The extent of involvement was also evaluated. Each lung was divided into three zones: upper (above the carina), middle (below the carina up to the inferior pulmonary vein), and lower (below the inferior pulmonary vein) zones. Each lung zone (total of six lung zones) was assigned a score that was based on the following: score 0, 0% involvement; score 1, less than 25% involvement; score 2, 25% to less than 50% involvement; score 3, 50% to less than 75% involvement; and score 4, 75% or greater involvement. The summation of scores provided the overall lung involvement score (maximal CT score for both lungs was 24).

### 2.4. Adverse Clinical Events

Composite events included death and requirements of extracorporeal membrane oxygenation (ECMO), mechanical ventilation, and high-flow nasal cannula (HFNC) oxygen therapy. The mechanical ventilation requirement included noninvasive ventilation and incubated/tracheotomy ventilation.

### 2.5. Ethics Approval

This report was approved by the West China Hospital Ethics Committee (2020–226). Oral informed consent was obtained from patients, and written informed consent was waived by the Ethics Committee for emerging infectious diseases.

### 2.6. Statistical Analysis

Data are expressed as the median and interquartile range (IQR (25th to 75th percentiles)) for continuous variables due to the skewed distribution (tested by the Shapiro–Wilk normality test) and as the count and percentage for categorical variables. The baseline demographics and clinical characteristics were compared between patients according to the plasma NT-proBNP levels at admission (<300 pg/ml or ≥300 pg/ml) using the Kruskal–Wallis test or the chi-squared test, as appropriate. A plasma NT-proBNP level <300 pg/ml was chosen as the cutoff value previously recommended for acute heart failure diagnosis [7]. The differences in NT-proBNP levels among PSI risk classes and CT score tertiles were tested using the Kruskal–Wallis test. Spearman's correlation coefficient was used to measure the association of the natural logarithm of NT-proBNP with the PSI and CT scores. Receiving operating characteristic (ROC) analysis was used to compare the predictive value of hs-cTnI, NT-proBNP, or the combination of these two biomarkers for the composite endpoint. cTnI and NT-proBNP entered the models as categorical variables but not as continuous variables. The cutoff point for cTnI was the 99th percentile upper reference limit (0.05 ng/ml), and that for NT-proBNP was 300 pg/ml. All statistical analyses were performed with IBM SPSS Statistics software (version 26.0) and Stata/SE software (version 15.1).

## 3. Results

### 3.1. Clinical Characteristics

A total of 91 (92.9%) patients were finally included in this study. The clinical characteristics of the patients are presented in Table 1. The median age of patients was 61 years (IQR, 47–69), and 39 (43.0%) of them were male. Hypertension was the most common coexisting illness (20.9%), and 10 patients (11.1%) had hs-cTnI values above the 99th percentile upper reference limit, which indicated acute myocardial injury. Twenty-three patients (25.3%) had NT-proBNP levels ≥300 pg/ml. White blood cell counts, plasma D-dimer levels, C-reactive protein levels, and hs-cTnI levels were higher in patients with NT-proBNP ≥300 pg/ml than in patients with NT-proBNP levels <300 pg/ml. The median NT-proBNP level at admission was 91.2 pg/ml (IQR, 30.0–305.4).

### 3.2. PSI and CT Scores

**Figure 2:**
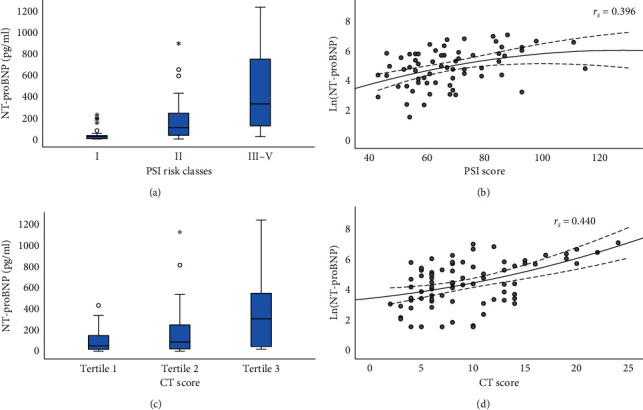
The box plots and the scatter plots of NT-proBNP levels with PSI classes or CT scores. (a) The box plot showing significant difference in NT-proBNP levels across PSI classes (*P* < 0.001). (b) The scatter plot showing the positive correlation between the natural logarithm of NT-proBNP level and PSI score (*r*_*S*_ = 0.396, *P*=0.001). (c) The box plot showing a significant difference in NT-proBNP levels across CT score tertiles (*P*=0.005). (d) The scatter plot showing that the natural logarithm of NT-proBNP level was significantly associated with CT score (*r*_*S*_ = 0.471, *P* < 0.001). NT-proBNP: N-terminal pro-B-type natriuretic peptide; PSI: pneumonia severity index; CT: computed tomography.

Patients with NT-proBNP levels ≥300 pg/ml had more fibrosis and pleural effusion according to imaging findings (NT-proBNP <300 pg/ml vs. NT-proBNP ≥300 pg/ml, 52.6% vs. 90.0%, *p*=0.003; 0.0% vs. 15.0%, *p*=0.016, respectively) (Table 1). According to the PSI, 26 (28.6%) patients were stratified into risk class I and 65 (71.4%) into risk classes II–V, for whom the quantitative PSI scores were also calculated (median, 66.0; IQR, 56.0–77.5). The NT-proBNP levels were significantly different across PSI risk classes (29.3 pg/ml (IQR, 9.0–44.2), 113.4 pg/ml (IQR, 39.8–270.5), and 336.3 pg/ml (IQR, 127.9–770.6), for classes I, II, and III–V, respectively; *p* < 0.001) (Figure 2(a)). The natural logarithm of the NT-proBNP level was positively correlated with the PSI score (*r*_*S*_ = 0.396, *p*=0.001) (Figure 2(b)). Furthermore, a total of 77 patients had high-resolution chest CT scans at admission. The median CT score was 8.0 (IQR, 6.0–13.0). The NT-proBNP levels were significantly different across CT score tertiles (55.1 pg/ml (IQR, 21.5–152.0), 91.2 pg/ml (IQR, 24.8–284.2), and 311.2 pg/ml (IQR, 47.4–564.1), for tertiles 1, 2, and 3, respectively; *p*=0.005) (Figure 2(c)). The natural logarithm of the NT-proBNP level was significantly associated with the CT score (*r*_*S*_ = 0.440, *p* < 0.001) (Figure 2(d)).

### 3.3. Clinical Outcomes

**Figure 3:**
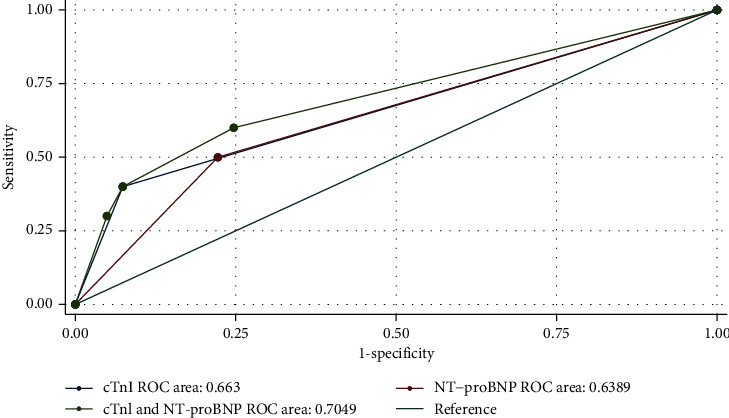
The receiving operating characteristic analysis comparing the predictive value of cTnI, NT-proBNP, or the combination of these two biomarkers for the composite events. NT-proBNP: N-terminal pro-B-type natriuretic peptide; hs-cTnI: high-sensitivity cardiac troponin I.

In total of 91 hospitalized patients, 2 cases of death (mortality rate: 2.3%) and 10 cases with composite events (events rate: 11.0%) were reported. Patients with NT-proBNP levels ≥300 pg/ml showed a potential risk for higher mortality (8.7% vs. 0%, *p*=0.062) and more composite events (21.7% vs. 7.4%, *p*=0.115) than patients with NT-proBNP levels <300 pg/ml (Table 2). The combination of cTnI and NT-proBNP seemed to show superior predictive value for the composite events compared with each individual component (ROC area: cTnI vs. NT-proBNP vs. the combination of cTnI and NT-proBNP, 0.66 (95% CI 0.50–0.83) vs. 0.64 (95% CI 0.47–0.81) vs. 0.70 (95% CI 0.52–0.89), respectively; *p*=0.299) (Figure 3).

## 4. Discussion

This study found that the plasma NT-proBNP level of COVID-19 patients was related to the severity of pneumonia. Patients with higher plasma NT-proBNP levels showed a potential trend for a higher risk of adverse clinical events. The combination of cTnI and NT-proBNP seemed to show superior predictive value for the composite events compared with each individual component.

The cardiovascular complications caused by coronavirus pneumonia outbreaks have previously highlighted the seriousness of this issue. In the 2003 SARS (severe acute respiratory syndrome) epidemic, researchers found that infected patients had complications such as hypotension, arrhythmia, diastolic dysfunction, and cardiac remodeling [8]. Pan et al. found that patients with severe SARS infection may experience cardiac arrest and even death [9]. Li et al. reported that echocardiography frequently demonstrates subclinical left ventricular diastolic impairment in SARS patients [10]. In the later MERS (Middle East respiratory syndrome) epidemic, some patients with this infection also had acute myocarditis and acute HF [11]. A recent study showed that 5 of 41 COVID-19 patients (12%) were diagnosed with a virus-related cardiac injury, which mainly manifested as a substantial increase in hypersensitive troponin I (hs-cTnI) (>28 pg/mL) [12]. Another study reported that, among 138 COVID-19 patients, 16.7% had arrhythmias and 7.2% had acute myocardial injury [2]. In this study, there were twice as many patients with NT-proBNP levels ≥300 pg/ml as there were patients with abnormal hs-cTnI values. This result indicated that cardiac dysfunction may be more common than cardiac injury in COVID-19 patients. A previous study reported that more than one-third of patients with pneumonia had transient left ventricular dysfunction, even in patients without a history of cardiac disease [3]. Elevated levels of interleukin (IL)-1*β*, IL-6, IL-12, interferon-inducible protein-10 (IP-10), and monocyte chemotactic protein-1 (MCP-1) can be found in some COVID-19 patients, which may be due to the systemic inflammatory storm resulting from an overaggressive host immune response to viral infection [12].

Cardiac dysfunction may be masked by pneumonia symptoms, which may delay treatment. Plasma concentrations of B-type natriuretic peptides increase during the acute phase of pneumonia, and the magnitude of this increase is associated with the severity and outcome of the infection [13]. Recently, Huang et al. reported that COVID-19 patients with cardiac injury always had higher BNP levels and higher hospital mortality than non-COVID-19 patients [14]. Thus, in severe cases of COVID-19, the measurement of NT-proBNP levels and the early monitoring of the possibility of HF may be helpful in the prevention and treatment of cardiac complications. Furthermore, our study revealed that increased concentrations of NT-proBNP in COVID-19 patients are more likely to result in worse clinical events.

Some limitations existed in the current study. First, this study is based on small sample size and a single-centre design, and bias would influence the results. Second, because of the limitations of medical resources at the onset of COVID-19, some patients lack electrocardiography data or echocardiography data, which limits the determination of the features and potential mechanisms of heart failure. Third, because the clinical observations of patients are still ongoing, many patients with and without cardiac dysfunction have not reached clinical endpoints. Further studies with larger population size and in multiple centres are warranted to investigate this issue in COVID-19 patients.

## 5. Conclusion

This study found that the plasma NT-proBNP level of COVID-19 patients was significantly related to the severity of pneumonia, indicating that HF needs to be assessed in this disease. The plasma test of NT-proBNP should be performed in COVID-19 patients to screen for heart dysfunction. This finding may help us identify COVID-19 pneumonia patients with coexisting HF, allowing for the implementation of earlier management strategies.

## Figures and Tables

**Table 1:** Characteristics of patients at baseline.

Characteristics	All patients (*n* = 91)	NT-proBNP <300 pg/ml (*n* = 68)	NT-proBNP ≥300 pg/ml (*n* = 23)	*P* value
Age, years	61.0 (47.0–69.0)	54.5 (41.5–67.0)	68.0 (66.0–75.0)	<0.001
Male, *n* (%)	39 (43.011)	27 (39.7)	12 (52.2)	0.296

Coexisting illnesses				
Hypertension, *n* (%)	19 (20.9)	8 (11.8)	11 (47.8)	<0.001
Diabetes, *n* (%)	4 (4.4)	3 (4.4)	1 (4.3)	1.000
Ischemic heart disease, *n* (%)	1 (1.0)	1 (1.5)	0 (0.0)	1.000
Chronic obstructive pulmonary disease, *n* (%)	7 (7.7)	3 (4.4)	4 (17.4)	0.043
Cancer	3 (3.3)	1 (1.5)	2 (8.7)	0.156

Vital signs				
Temperature, °C	36.5 (36.4–36.7)	36.5 (36.4–36.7)	36.6 (36.4–36.7)	0.712
Respiratory rate, per min	20.0 (18.0–21.3)	20.0 (18.0–21.0)	20.0 (18.0–22.0)	0.323
Heart rate, beats per min	86.5 (78.0–98.0)	88.0 (78.0–98.0)	82.0 (76.0–98.0)	0.289
Systolic blood pressure, mmHg	128.5 (120.0–140.0)	125.0 (119.0–135.0)	135.0 (125.0–47.0)	0.034
Diastolic blood pressure, mmHg	78.0 (73.5–84.5)	77.0 (73.0–83.0)	80.0 (75.0–90.0)	0.211

Laboratory findings				
White cell count, per mm^3^	5300 (4000–6700)	4945 (3773–6180)	6200 (5280–7390)	0.003
Hemoglobin, g/liter	125.0 (114.0–137.0)	124.5 (114.0–35.5)	128.0 (114.0–137.0)	0.654
Hematocrit	0.36 (0.33–0.39)	0.36 (0.32–0.39)	0.37 (0.33–0.39)	0.638
Platelet count, per mm^3^	222,000 (122,000–282,000)	227,000 (170,000–284,500)	199,000 (132,000–273,000)	0.300
D-dimer	0.70 (0.35–1.83)	0.47 (0.37–1.45)	1.79 (0.83–8.88)	<0.001
CD4+ cell	444 (258–586)	468 (303–610)	275 (143–521)	0.018
CD8+ cell	244 (156–378)	252 (167–405)	216 (93–340)	0.149
Creatinine, *μ*mol/liter	59.0 (48.0–71.0)	57.0 (48.0–69.8)	66.0 (49.0–76.0)	0.113
Alanine aminotransferase, U/liter	22.0 (15.0–39.0)	20.0 (14.3–35.8)	27.0 (18.0–44.0)	0.038
Aspartate aminotransferase, U/liter	24.0 (18.0–34.0)	22.0 (17.0–30.8)	33.0 (24.0–44.0)	0.004
Albumin, g/liter	37.8 (34.4–41.3)	38.3 (36.0–41.6)	35.7 (31.9–37.5)	0.005
Sodium, mEq/L	142 (138–145)	142.0 (139.0–145.0)	143.0 (37.0–145.0)	0.916
Procalcitonin, ng/mL	0.04 (0.03–0.07)	0.04 (0.03–0.06)	0.07 (0.03–0.14)	0.074
C-reactive protein, mg/liter	11.2 (5.0–46.8)	8.1 (5.0–38.8)	55.0 (5.6–169.5)	0.005
Creative kinase MB, U/liter	1.0 (0.6–1.5)	0.92 (0.58–1.35)	1.16 (0.94–1.98)	0.002
hs-cTnI > 99th percentile URL	10 (11.1)	3 (4.4)	7 (30.4)	0.002
NT-proBNP, pg/ml	91.2 (30.0–305.4)	49.8 (23.1–28.1)	437.7 (346.7–808.1)	<0.001

Radiographic finding				
More than two lobes involvement	76 (98.7)	56 (98.2)	20 (100.0)	1.00
Pleural effusion	3 (3.9)	0 (0.0)	3 (15.0)	0.016
Ground-glass opacity	74 (96.1)	55 (96.5)	19 (95.0)	1.00
Consolidation	56 (72.7)	40 (70.2)	16 (80.0)	0.396
Fibrosis	48 (62.3)	30 (52.6)	18 (90.0)	0.003
CT score	8.0 (6.0–13.0)	7.0 (5.0–10.5)	14.0 (8.3–19.0)	<0.001

Pneumonia severity index (PSI)				
Class I	26 (28.6)	26 (38.2)	0 (0.0)	<0.001
Class II	43 (47.3)	33 (48.5)	10 (43.5)	
Classes III–V	22 (24.2)	9 (13.2)	13 (56.5)	

Total point score for PSI class > I	66.0 (56.0–77.5)	61.0 (54.0–70.0)	73.0 (63.0–87.0)	0.005

*Note.* Data are expressed as median (interquartile range) or counts and percentage, as appropriate. hs-cTnI: high-sensitivity cardiac troponin I; URL: upper reference limit; NT-proBNP: N-terminal pro-B-type natriuretic peptide; CT: computed tomography.

**Table 2:** Clinical events across plasma NT-proBNP groups.

Clinical events	All	NT-proBNP <300 pg/ml	NT-proBNP ≥300 pg/ml	*P* value
	(*n* = 91)	(*n* = 69)	(*n* = 23)	
HFNC oxygen therapy	2 (2.2)	1 (1.5)	1 (4.3)	0.444
Mechanical ventilation	8 (8.8)	4 (5.9)	4 (17.4)	0.108
ECMO requirement	2 (2.2)	1 (1.5)	1 (4.3)	0.444
All-cause death	2 (2.2)	0 (0)	2 (8.7)	0.062
Composite events^*∗*^	10 (11.0)	5 (7.4)	5 (21.7)	0.115

*Note.* Data are expressed as counts and percentage. Pearson's chi-squared or Fisher's exact test was used to test the difference between NT-proBNP groups, as appropriate. ^*∗*^The composite events were the composite of HFNC oxygen therapy, mechanical ventilation, ECMO requirement, or all-cause death. NT-proBNP: N-terminal pro-B-type natriuretic peptide; HFNC: high-flow nasal cannula; ECMO: extracorporeal membrane oxygenation.

## Data Availability

The data used to support the findings of this study are available from the corresponding author upon request.

## Supplementary Materials

Supplementary Materials Appendix I. Figure S1. Flowchart for the pneumonia severity index.
