# Supplementary material for: Association between NT-proBNP Level and the Severity of COVID-19 Pneumonia
Source: Cardiol Res Pract. 2021 Jul 8;2021:5537275. doi: 10.1155/2021/5537275 (PMC8266470; doi:10.1155/2021/5537275)
Supplement: Supplementary Materials — Appendix I. Figure S1. Flowchart for the pneumonia severity index. [file 5537275.f1.docx]

**Supplementary Files**

This Supplementary Files has been provided by the authors to give readers additional about their work.

**Appendix I. Figure S1.** Flow chart for the Pneumonia Severity Index

**

**
